# Treatment Approaches for Interoceptive Dysfunctions in Drug Addiction

**DOI:** 10.3389/fpsyt.2013.00137

**Published:** 2013-10-18

**Authors:** Martin P. Paulus, Jennifer L. Stewart, Lori Haase

**Affiliations:** ^1^Department of Psychiatry, University of California San Diego, La Jolla, CA, USA; ^2^Psychiatry Service, VA San Diego Healthcare System, La Jolla, CA, USA

**Keywords:** addiction, interoception, exercise, meditation, insula

## Abstract

There is emerging evidence that individuals with drug addiction have dysfunctions in brain systems that are important for interoceptive processing, which include, among others, the insular and the anterior cingulate cortices. These individuals may not be expending sufficient neural resources to process perturbations of the interoceptive state but may exert over-activation of these systems when processing drug-related stimuli. As a consequence, insufficient detection and processing of interoceptive state changes may result in inadequate anticipation and preparation to adapt to environmental challenges, e.g., adapt to abstinence in the presence of withdrawal symptoms. Here, we integrate interoceptive dysfunction in drug-addicted individuals, with the neural basis for meditation and exercise to develop a heuristic to target the interoceptive system as potential treatments for drug addiction. First, it is suggested that mindfulness-based approaches can modulate both interoceptive function and insular activation patterns. Second, there is an emerging literature showing that the regulation of physical exercise in the brain involves the insula and anterior cingulate cortex and that intense physical exercise is associated with a insula changes that may provide a window to attenuate the increased interoceptive response to drug-related stimuli. It is concluded that the conceptual framework of interoceptive dysfunctions in drug addiction and the experimental findings in meditation and exercise provide a useful approach to develop new interventions for drug addiction.

## Interoceptive Dysfunction in Drug Addiction

Interoception comprises receiving, processing, and integrating body-relevant signals together with external stimuli to affect motivated behavior (1, 2). This process fundamentally affects the degree to which individuals approach or avoid drugs of abuse (3). Different conceptualizations of interoception have included its definition as the state of the individual at a particular point in time (4), or as the sensing of body-related information in terms of awareness (5), sensitivity (6), or accuracy of the sensing process (7). Interoception provides an anatomical framework for identifying pathways focused on modulating the internal state of the individual. This framework comprises peripheral receptors (7), c-fiber afferents, spino-thalamic projections, specific thalamic nuclei, posterior and anterior insula as the limbic sensory cortex, and anterior cingulate cortex (ACC) as the limbic motor cortex [for reviews, see Ref. (8, 9)]. Central to the concept of interoception is that body-state relevant signals comprise a rich and highly organized source of information that affects how an individual engages in motivated behavior. Importantly, interoception is linked to homeostasis (10), which implies that an individual’s motivated approach or avoidance behavior toward stimuli and resources in the outside world is aimed at maintaining an equilibrium. For example, a person will approach a heat source in a cold environment but will avoid it when the ambient temperature is high.

The insular cortex is a complex brain structure, which is organized macroscopically along an anterior-posterior (1) and superior-inferior axis (11) and microscopically as granular, dysgranular, and agranular from posterior to anterior insula, respectively (12, 13). The anterior cluster is predominately activated by effortful cognitive processing, whereas the posterior region is mostly activated by interoception, perception, and emotion (14). Moreover, the anterior insula, potentially together with the ACC, appears to pivotally influence the dynamics between default-mode and executive control networks (15). The insula is thought to be the central nervous system hub for interoceptive processing such that somatosensory relevant afferents enter the posterior insula and are integrated with the internal state in the mid-insula, and re-represented as complex feeling states within the anterior insular cortex. Although there has been some debate, a recent meta-analysis suggests that the anterior insula is critical and necessary for emotional awareness (16).

The ACC has been labeled the limbic motor cortex by some (9) for review, see Ref. (17), and is thought to be the critical interface between cognitive and emotion processing (18). In particular, Von Economo neurons, which are projection neurons located in layer V within the ACC and frontoinsular cortex, have been implicated in the integrative function of the ACC (19). However, whether different parts of the ACC are involved in distinct processes and whether these processes are segregated for different functions is still highly debated. On the one hand, several investigators have proposed an anatomically based topography of the ACC consisting of subgenual, pregenual, and anterior mid-cingulate cortex, which are cytoarchitecturally distinct and have different connectivity with other brain structures (20). In particular, whereas the rostral ACC (comprising both sub- and pregenual ACC) is important for emotional processing, the dorsal or mid-cingulate cortex is thought to implement cognitive control and emotion regulation (21). However, there is also considerable overlap between the “cognitive” division of the ACC and the mid-cingulate area that processes pain and fear (20). This overlap is consistent with the idea that dorsal-caudal regions of the ACC and medial prefrontal cortex are involved in appraisal and expression of negative emotion (22). On the other hand, based on a meta-analysis of imaging studies, some investigators have proposed that negative affect, pain, and cognitive control activate an overlapping region within the anterior mid-cingulate cortex, which can be thought of as a hub that links reinforcers to motor centers responsible for expressing affect and executing goal-directed behavior (23). In particular, it has been proposed that the ACC supports the selection and maintenance of extended, context-specific sequences of behavior directed toward particular goals that are learned through a process of hierarchical reinforcement learning (24). This generalized view of ACC functioning is consistent with the proposal that this structure, among other functions, orchestrates approach or avoidance behaviors in response to particular internal body states that involve homeostatic perturbations (25). This function of the ACC is supported by the strong functional (26) and anatomical (27) connections between the anterior insula and the ACC. This systemic view is also aligned with a prediction error based conceptualization of the specific computational processes that may be carried out within this structure. For example, a special population of neurons in the ACC seems to fire with positive and negative value information during the decision phase using a unified encoding scheme, which is based on reward prediction errors (28). Others have argued that the ACC encodes salient prediction errors for appetitive and aversive stimuli (29). Finally, activation in the rostral ACC correlates with a belief-based prediction error, which is driven by the discrepancy between the anticipation and action of others (30). Taken together, the ACC receives information about the individual’s current state as well as the expected state, and computes various types of error signals that help to establish the selection of an action that is optimally adapted to the higher order goal state.

We have previously hypothesized that individuals who are at risk for drug addiction or who have developed addiction show altered interoceptive processing (31). In particular, individuals who show attenuated processing of internal body states may be at higher risk for substance dependence because these individuals are not able to utilize “body states” to guide their decision-making (32). There has been some evidence that neural substrates processing body-relevant information and their associated neural circuits play an important role in drug addiction (33, 34). Individuals take substances to feel better or to avoid feeling worse. The positive and negative reinforcing aspects of drugs of abuse have given rise to a tremendous insight into the behavioral processes (35, 36), neural systems (37, 38), and molecular mechanisms (39, 40) of drug addiction. In comparison, the interoceptive framework is much less developed and needs further empirical validation. We have conceptualized that an individual’s motivation to approach or avoid a stimulus, including drugs of abuse, results from a brain-generated body prediction error (31, 41–43), i.e., the difference between the experienced and the expected internal state of the individual. In particular, we have argued more recently that optimal behavior emerges from a computational process involving probabilistic representation of belief states in the context of partially observable Markov decision processes (44). In this context, body prediction error is evaluated within the context of the individual’s belief about external stimuli and their relevance for specific outcomes. For example, when considering a choice of engaging in risky behavior, individuals with substance use disorders may not appropriately engage the insular cortex to signal the potential aversive outcome. On the other hand, seeing a cue that has previously been associated with drug-taking behavior may generate a large insula response and provide overwhelming approach behavior manifested in cravings and the urge to use.

There is emerging evidence of insula dysfunction across different groups of substance dependent individuals. Yet, it is important to not engage in excessive inverse inference and conclude that because there is evidence for insula dysfunction, this dysfunction extends to interoceptive processing. It is more likely that interoception is one among various processes that are altered in these populations. Specifically, exposure to nicotine-related stimuli increases blood flow in a large network, including the insular cortex (45) and smokers when not smoking (46) and when anticipating to smoke (47) show greater anterior insula activation. In addition, recently abstinent smokers who were more likely to relapse also showed greater insula and ACC activation to smoking-related images (48). Finally, smokers with higher levels of nicotine dependence showed enhanced insula reactivity to smoking-related pictures (49). Similarly, acute administration of cannabis increases blood flow (50) and functional magnetic resonance imaging (fMRI) perfusion signal (51, 52) in the insula. Several fMRI studies demonstrate that cannabis users exhibit less activation in the insula during inhibitory processing (53), which has been linked to reduced error awareness (54) but, in contrast, show enhanced insular response to cannabis-related cues (55). In amphetamine users, fMRI studies show attenuated insula activation in cognitive control (56) and emotion processing tasks (57) but enhanced response to pharmacological agents, such as modafinil, aimed at increasing cognitive control (58). Moreover, within an amphetamine dependent sample, attenuated insula activation during a simple decision-making task was associated with increased propensity for relapse (59). Similar to amphetamine, there is evidence for dysfunctional insula in cocaine dependent individuals. fMRI research demonstrates attenuated insula activation during an inhibitory task (60) but enhanced insula response in other tasks such as those involving monetary reward-related processing (61), stress-related imagery (62), presentation of cocaine-related cues (63), which is related to the degree of craving (64, 65). This altered insula reactivity in these individuals may undergo dynamic changes as a function of sobriety, e.g., longer periods of abstinence in cocaine dependent subjects relative to those with fewer days of sobriety showed attenuated insula responses during errors on a cognitive control task (66). Considering the different substance dependent populations, there is consensus that insula reactivity is reduced during cognitive control tasks but enhanced when individuals are exposed to cues or processes involving reward. This view is consistent with that expressed by Garavan (67), who stated that “drug craving may be an example of the anterior insula’s role in interoception and subjective feeling states,” which is influenced by changes in general internal states such as satiety (68) as well as top-down cognitive modulation. Moreover, these findings are consistent with the notion that cue reactivity involves a significant visceral component and an urge to act to acquire the drug. In summary, insula dysfunction and altered interoceptive processing consisting of either attenuated processing of non-drug stimuli and excessive processing of drug-related stimuli is emerging as an important pathological process in addiction (33, 69).

## Modulating Interoceptive Systems

There are several possible approaches to modulate how an individual processes and integrates afferent sensing from the inside of the body. The basic proposition is that altering these processes will affect the way an individual processes drug-related cues due to their significant effect on the body state. Here, we will focus on two strategies, that have been used and for which there is some empirical evidence for their efficacy in treating drug addiction. First, we will discuss mindfulness approaches, which are aimed at creating a non-judgmental awareness of the experiences within the body as a function of events that take place in the person’s life. Second, we will briefly review the role of physical exercise, which creates an acute bottom up perturbation of interoceptive processing.

### Mindfulness approaches

Mindfulness is “the awareness that emerges through paying attention on purpose, in the present moment, and non-judgmentally to the unfolding of experience moment by moment” (70). Mindful awareness is cultivated by providing guided instruction in mindfulness meditation practices including breath-focused attention and body-scanning of sensory experiences. Mindfulness-based techniques, and in particular, mindfulness-based stress reduction (MBSR) developed by Jon Kabat-Zinn (71, 72), have been shown to reduce stress-related sequelae (e.g., self-reported stress, medical symptoms, neuroendocrine changes) associated with chronic mental health disorders [e.g., Ref. (73, 74)], medical conditions [e.g., Ref. (75, 76)], and non-clinical populations [e.g., Ref. (77–79)]. As part of mindfulness training, individuals are trained to focus their attention and are instructed to return their attention to their focus point when they become distracted. Thus, it is not surprising that individuals who undergo meditation training show improved attention processing (80–83). There is converging evidence that anatomical (84–87) and functional (88–93) brain changes are associated with mindfulness training, particularly in the insula, ACC, and other brain structures such as the prefrontal cortex. In particular, during pain stimulation, experienced meditators show an increased reactivity consisting of low baseline activity coupled with high response in the anterior insula, which was related to accelerated habituation within the amygdala (94). Experienced meditators also show greater gray matter concentration in the anterior insula (86), which may be the consequence of attention-related adaptation. Others have shown that degree of mindfulness training was related to more efficient pain processing (93), greater inhibitory control (95), greater interoceptive attention in anterior dysgranular insula, as well as altered functional connectivity between posterior insula and dorsomedial prefrontal cortex (96). Taken together, training involving attention modulation and interoception increases the efficiency of the insula and associated neural systems when processing afferent information.

A growing literature suggests that mindfulness-based approaches for the treatment of substance use disorders may be able to reduce the susceptibility to relapse. From a mechanistic perspective, these approaches are intended to increase discriminative awareness, with a specific focus on acceptance of uncomfortable states or challenging situations without reacting with habitual affect (97). There is a small number of well-designed clinical trials and experimental laboratory studies of mindfulness approaches in smoking, alcohol dependence, and illicit substance use (98). For example, meditation has been used as an effective adjunctive therapy for relapse prevention in alcohol dependence (99), smoking cessation (100), and a diverse group of substance dependent individuals (101). In these trials, individuals participating in mindfulness-based interventions demonstrated significantly lower rates of substance use and greater decreases in baseline and negative affect-induced craving (97).

Despite the evidence for the clinical efficacy of mindfulness-based methods, the specificity of the underlying cognitive and neural mechanisms is still unclear (102). For example, increased levels of mindfulness were associated with lower alcohol attentional bias, stress, and craving, as well as greater alcohol-related self-efficacy (103). Neuroimaging studies indicate greater dorsolateral prefrontal cortex responses during executive processing (95) and decoupling of functional connectivity between subgenual ACC and insula when viewing craving-inducing stimuli such as smoking pictures (104). Finally, there is evidence that cue-elicited high-frequency heart rate variability may be modulated by mindfulness and may function as a peripheral marker for relapse susceptibility (105). On the whole, the effect of mindfulness appears to involve brain systems that are important for interoceptive processing in general, and relapse in particular, and alters peripheral markers that have been associated with interoceptive processing. Specifically, mindfulness may enhance one’s ability to adequately process body-state relevant information, i.e., improve insula recruitment when experiencing changes in interoceptive afferents, without having to select actions, i.e., engage the ACC to recruit approach or avoidance behaviors. The relative “disconnect” between sensing and acting might result in short-term relief such that following mindfulness intervention, an individual may be able to recognize feelings of craving without acting on them. In other words, the disengagement of motivated action as a result of interoceptive perturbation may enable the individual to learn new actions and not engage in habitual drug use behavior.

### Exercise

There is a growing interest in understanding the neural processes underlying physical exercise in general and its role in optimizing levels of physical performance. Several investigators have begun to delineate which brain processes contribute to athletic performance (106, 107). The insular cortex has been identified as a component of the so-called “central governor,” i.e., the brain systems that are important for modulating the degree to which individuals engage in demanding athletic performance (106, 108). Specifically, increased insular regional cerebral blood flow (rCBF) was observed during active, but not passive, cycling (109). Furthermore, both the insula and ACC were also found to activate during imagined exercise (110). Finally, greater insular rCBF was positively correlated with levels of perceived cycling intensity (111) and with individual blood pressure changes.

The central governor model is a conceptual approach to determine how interoceptive afferents influence levels of performance. In particular, the model focuses on perceived exertion (112), i.e., the subjective perception of exercise intensity, as a function of ongoing exercise (113). Recently this model has been extended (114) to include a system of simultaneous efferent feed-forward and afferent feedback signals that are thought to optimize performance by overcoming fatigue through permitting continuous compensation for unexpected peripheral events (115). Afferent information from various physiological systems and external or environmental cues at the onset of exercise can be used to forecast the duration of exercise within homeostatic regulatory limits. This enables individuals to terminate the exercise when the maximal tolerable perceived exertion is attained. In this model, the brain creates a dynamic representation of an expected exertion against which the experienced exertion can be continuously compared (114) to prevent exertion from exceeding acceptable levels. Moreover, the notion of a differential between expected and experienced exertion parallels our model of the body prediction error (42). For example, an athlete may utilize the experience of heavy breathing or heart rate to adjust effort in the presence of external stimuli signaling an upcoming increase in demand. However, Marcora and colleagues have argued that perceived exertion is generated by a top-down or feed-forward signal (107), i.e., the brain – not the body – generates the sense of exertion and proposed that the a centrally generated corollary discharge of the brain is critical for optimal effort (116). Moreover, it has been argued that mental fatigue affects performance via altered perception of effort rather than afferent and body originating cardiorespiratory and musculoenergetic mechanisms (117). In summary, it is most likely the brain, not the body, that sets the subjective level of perceived exertion as a consequence of an interaction between feed-forward (expectations) and feedback (body-relevant sensing) information, which maintains a homeostatic state for the individual to be resilient to physical perturbations. This theoretical formulation is analogous to the perturbation experienced by a drug dependent individual when experiencing craving due to conditioned stimuli that predict availability of drug.

There is an emerging literature on the efficacy and mechanisms of exercise in substance dependent individuals. As recently reviewed in (118), the beneficial effects of exercise as an adjunct in treatment of substance use disorders may be due to its ability to facilitate dopaminergic transmission, normalize glutamatergic and dopaminergic signaling, and reverse drug-induced changes in chromatin via epigenetic interactions with brain-derived neurotrophic factor (BDNF) in the reward pathway (118). Acute exercise reduces alcohol urges (119), cigarette cravings (120, 121), and daily cannabis use (122). Neuroimaging studies have shown that relative to a resting condition, individuals undergoing exercise showed reduced desire to smoke and attenuated brain activation in limbic areas in response to smoking-related stimuli (120) and an accompanying increase in default-mode activation (123). However, the precise cognitive and neural mechanisms that contribute to the beneficial effects of exercise on drug-taking behavior in individuals with substance use disorder still await further study. One possible hypothesis is that the ACC, via repeated engagement of controlled goal-directed action, is better prepared to respond to body-relevant information that is initiated by drug-relevant stimuli. Thus, in some ways, exercise might also alter cognitive control mechanisms that are important for drug addiction.

## Neural Basis of Interoceptive Plasticity and Treatment in Drug Addiction

Interoception, particularly its dysfunction in individuals with drug addiction, provides a conceptual and neural systems framework as well as various experimental approaches to examine the mechanisms underlying interventions that may be effective in reducing an individual’s susceptibility to drug use, cravings associated with exposure to conditioned stimuli, and the ability to select alternative behaviors when anticipating aversive states associated with substance withdrawal. One way to conceptualize the degree of motivated approach/avoidance behavior in the context of drug addiction is to view the emerging behavior as a consequence of a homeostatic adjustment to a body prediction error (31, 41–43), i.e., the difference between the experienced and the expected internal state of the individual. However, the simple difference between an experienced and expected body state does not explain opposing insular cortex effects in different task settings in substance use populations. We have proposed that optimal behavior emerges from a computational process involving probabilistic representation of belief states (44). Here, body prediction error is evaluated within the context of the individual’s belief about external stimuli and their relevance for specific outcomes. Specifically, seeing a drug-related cue may generate a large insula response and provide an overwhelming urge to engage in drug seeking behavior. This view of the differential contribution of the interoceptive afferents to motivated behavior is consistent with the multi-modal connections of the insula with other brain areas. These modulatory influences may ultimately determine whether an “embodied” state is (a) amplified and experienced, (b) contributes to ongoing behavior, and (c) becomes a target for the cognitive control system to modulate its influence. This conceptual approach, although based on an interoceptive heuristic, also involves computational processes that are clearly within the realm of cognitive control, e.g., the facilitation or inhibition of competing responses. Thus, this approach is not inconsistent with the notion of cognitive control dysfunctions in addiction (124, 125) and should be seen as complementary, i.e., similar to the notion of embodied cognition (126).

Both meditation and exercise can be viewed as systematic approaches to alter the way approach/avoidance behavior emerges from a body prediction error. For example, it appears that elite athletes, which have undergone extensive physical exercise have the ability to use predictive signals to modulate the insula response to an aversive interoceptive perturbation (127). Similarly, others have shown that meditation alters the influence of the dorsomedial prefrontal cortex on insula activation in response to an interoceptive awareness task (96). Together, these findings point toward the plasticity of the neural substrates that are important for interoceptive processing. Practically speaking, meditation and exercise may modulate the interoceptive circuitry by altering the way the individual processes stimuli that are predictive of an altered homeostasis, e.g., smoking cues or the emergence of a substance withdrawal state, and enables one to engage in alternative behaviors. Moreover, exercise helps to optimize complex goal-directed behaviors, which may train the ACC to computer more appropriate value signals. Meditative approaches highlight the influence of pre-existing belief systems on the evaluation of conditioned stimuli and may thereby alter the computational process involving probabilistic representation of belief states as proposed previously (44). Practically, this may optimize behavioral choices in the context of goal states that are associated with an immediate reward but long-term aversive consequences. Nevertheless, future experimental approaches will need to examine whether this conceptualization adequately describes the restricted behavioral repertoire observed in individuals with substance use disorders. Finally, many open questions remain regarding the mechanisms of meditation and exercise on the neural systems that are important for recovery from substance use disorders. Overall, the conceptual framework of interoceptive dysfunctions in drug addiction and the experimental findings in meditation and exercise provide a useful approach to use neuroscience to develop new interventions for drug addiction.

## Conflict of Interest Statement

The authors declare that the research was conducted in the absence of any commercial or financial relationships that could be construed as a potential conflict of interest.
